# Optical Impact of Corneal Clearance in Healthy Eyes Fitted with Scleral Contact Lenses: A Pilot Study

**DOI:** 10.3390/jcm11123424

**Published:** 2022-06-14

**Authors:** María Villa, Francisco Cavas, David P. Piñero

**Affiliations:** 1 Department of Optics, Pharmacology and Anatomy, University of Alicante, 03690 Alicante, Spain; mariavillag3@gmail.com; 2 Department of Structures, Construction and Graphic Expression, Technical University of Cartagena, 30202 Cartagena, Spain; francisco.cavas@upct.es

**Keywords:** scleral contact lens, central corneal clearance, optical quality, refraction, high order aberration

## Abstract

This pilot study was conducted to evaluate the effect on refraction and optical quality of the increase in the corneal clearance after fitting a specific model of scleral contact lens (ScCL) in healthy subjects. A total of 15 eyes from 15 subjects were enrolled in the study, with evaluation of refraction, ocular aberrations and central corneal clearance with the same model of ScCL (ICD Toric, Paragon Vision Science, Gilbert, AZ, USA), but using 3 different sagittal heights: 4200, 4500 and 4800 µm. Mean values of corneal clearance for each ScCL fitted were 418.1 ± 112.1, 706.5 ± 120.3 and 989.9 ± 117.0 µm, respectively. Significant changes were detected in the spherical equivalent and high-order aberrations, especially coma and spherical aberration, when fitting ScCLs of increasing sagittal heights compared to the pre-fitting values. In conclusion, the increase in central corneal clearance when fitting ScCLs affects refraction, leading to a more myopic refractive error, and inducing an increase in different ocular HOAs. This should be considered when fitting ScCLs, especially multifocal designs.

## 1. Introduction

Scleral contact lenses (ScCL) are contact lenses made of rigid, gas-permeable materials with a large diameter that completely bear on the sclero-conjunctival surface [1]. Thus, a tear reservoir between the contact lens and the corneal surface is created, which is maintained over hours of use [2]. The lacrimal reservoir is known as corneal clearance, central corneal clearance or vault, and is the responsible for the excellent optical quality provided by ScCLs, as corneal irregularities are largely neutralized by this corneal clearance generated [2]. For this reason, ScCLs are mainly fitted in cases of irregular cornea [3,4,5], such as corneal ectasias, keratoplasty, or post-corneal trauma, allowing one to correct the different refractive errors and high order aberrations that are present in these cases. Furthermore, ocular surface diseases are another indication for ScCL fitting [3,4,5,6], such as Sjögren’s syndrome or dry eye [7], having this tear reservoir a therapeutic effect.

Although ScCL designs from different manufacturers may differ in overall diameter, all ScCLs have the same basic lens geometry: optical zone, transition zone, and landing zone [2]. The ScCL with toric geometry is characterized by presenting a bearing zone with toricity, improving the stability of the lens on the ocular surface, and not interfering with the central zone of the lens [8]. One of the critical factors of ScCL fitting is the selection of the corneal clearance target, as it should avoid any corneal touch during lens wear but allow enough oxygen to reach the cornea. A previous study using a ScCL of 18 mm of diameter with 2 different central clearances of 200 and 400 µm has demonstrated that the cornea presents oxygen levels lower than those necessary to prevent hypoxia while wearing the lenses [9]. In addition, this study stated that a clearance of 400 µm should be avoided since there is a 30% reduction in oxygen compared to the 200 µm clearance [9]. Specifically, to minimize the potential corneal hypoxia induced by the ScCL wear, Michaud et al. [10] recommended the use of ScCLs made of high Dk materials (Dk > 150), a central lens thickness of 250 µm and targeting a corneal clearance not exceeding 200 µm. In addition, another study carried out by Compañ et al. [11] stated that the Dk of the ScCL manufacturing material must present a value of at least 125 to avoid the development of a clinically significant edema, the value of the central thickness of the contact lens must not be greater than 250 µm and the value of the corneal clearance thickness must be less than 150 µm.

In general, in ScCL fittings, a clearance of 200–300 µm is considered adequate [2], being this clearance, a factor potentially influencing the corneal shape and power, but not inducing a clinically significant corneal edema [12]. Despite the published studies on the impact on oxygen transmissibility of different clearances used with ScCLs [9,10,11], few studies have been conducted to evaluate the impact on refraction and high order aberrations and consequently on visual quality [13,14]. The aim of this study was to evaluate in a pilot study the effect on refraction and ocular high order aberrations of the increase in the corneal clearance with a specific model of ScCL in healthy subjects.

## 2. Materials and Methods

### 2.1. Patients

A pilot pre-post pseudo-experimental study including 15 participants with ages ranging from 18 to 35 years was conducted. Only one eye was randomly selected from each participant, trying to avoid the potential bias due to the correlation between the two eyes of the same participant. All of the measurements were carried out at the Optometric Clinic of the University of Alicante. The study was approved by the Ethics Committee of the Hospital General Universitario of Alicante (CEIm PI2020-048, ISABIAL 200045). This study was performed in accordance with the ethical standards laid down in the 1964 Declaration of Helsinki and its subsequent modifications. Participants previously signed an informed consent, being informed about the nature of the study before their evaluation.

Inclusion criteria were healthy eyes with spherical refractive error between −10 and +10 D and cylinder of less than 3.00 D, and best corrected visual acuity (BCVA) of 0.0 log MAR or better. Exclusion criteria were any ocular pathology, active ocular treatment at the time of the study, active systemic disease, any previous ocular surgical intervention and being ScCL wearer. In the case of contact lens wearers, the discontinuation of the use of these contact lenses was indicated at least 15 days for soft contact lenes and 30 days for rigid contact lenses before the examination.

### 2.2. Contact Lens Fitting

All subjects were fitted and evaluated afterwards with three ScCLs of the same model of scleral lens, ICD Toric (Paragon Vision Science, Gilbert, AZ, USA). This scleral lens has an overall diameter of 16.5 mm and a central thickness of 300 µm (Paflufocon D material, Dk = 100 barrier). Specifically, 3 different lenses from the ICD testing box (ICD, Family of lens designs) were selected and fitted following this established order:

- Lens 1: sagittal height 4200 μm, lens power −2.00 D, scleral toricity 125 µm, base curve 7.67 mm (base curve 44.00 D);
- Lens 2: sagittal height 4500 μm, lens power −5.00 D, scleral toricity 125 µm, base curve 6.89 mm (base curve 49.00 D);
- Lens 3: sagittal height 4800 μm, lens power −8.00 D, scleral toricity 125 µm, base curve 6.61 mm (base curve 51.00 D).

### 2.3. Examination Protocol

All of the participants underwent a complete eye examination including manifest refraction, measurement of best corrected visual acuity (BCVA), slit lamp biomicroscopic analysis, topographic examination using the Pentacam HR system (Oculus Optikgeräte GmbH, Wetzlar, Germany) and anatomical and optical analysis of the eye with the multidiagnostic platform Visionix VX120 (Luneau Technologies, Chartres, France) that allowed the measurement of parameters such as anterior chamber depth (ACD), central corneal thickness (CCT), corneal white-to-white distance (WTW) parameter, keratometry (K), ocular high order aberrations (HOA) (5-mm pupil aperture) and spherocylindrical refraction. These same examinations were performed 10 min after fitting the 3 ScCLs used in the current study. Once the measurements were performed with each contact lens, it was removed and a resting period of 30 min was waited until the next fitting, with instillation of artificial tears each 5 min to perform a wash-out of the ocular surface.

**Figure 1:**
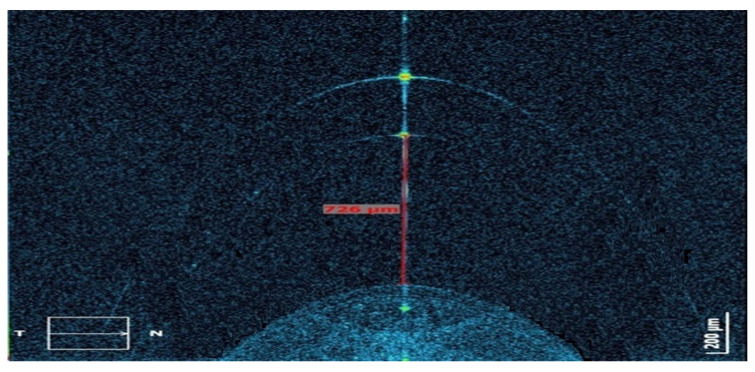
Measurement procedure of the central corneal clearance with the different scleral contact lenses (ScCL) fitted using the Fourier-domain optical coherence tomography (OCT) system Copernicus HR (Optopol Technology Sp. Z.o.o, Zawiercie, Poland).

All of the lenses were fitted and all of the measurements were performed by the same professional (MV). For the insertion, the ScCL was filled with saline solution that was stained with a fluorescein strip (Fluorescein paper, Haag-Streit AG, Köniz, Switzerland) to facilitate the evaluation of the fluorogram. The thickness of the central corneal clearance with each of the lenses was evaluated with the Fourier-domain optical coherence tomography (OCT) system Copernicus HR (Optopol Technology Sp. Z.o.o, Zawiercie, Poland). Images were taken on the horizontal axis and in the center of the pupil using the 5-line module that the instrument has. Corneal clearance values were manually determined using the caliper tool of the system 10 min after the fitting (Figure 1). Three consecutive measures of this clearance were obtained, and the mean value was used for the final analysis.

### 2.4. Statistical Analysis

The SPSS 24.0 statistical software (IBM SPSS Statistics, IBM Armonk, NY, USA) was used for the statistical analysis. Normality of the data distribution for all of the variables analyzed was evaluated using the Kolmogorov-Smirnov test. The Friedman test was used to assess the significance of differences in refraction and aberrometric parameters with the three different ScCLs fitted, since the data samples were not normally distributed. *p*-values of less than 0.05 were considered as statistically significant. Non-parametric Spearman correlation coefficients were calculated to assess the relationship between the changes in ocular refraction and aberrometry with the three different corneal clearances obtained with the three ScCLs fitted. Sphero-cylindrical refraction measurements were converted into a vector notation using the power vectors described by Thibos and Horner [15].

## 3. Results

The sample of this study included 15 eyes of 15 patients ranging in age from 18 to 32 years old (mean age: 24.5 ± 3.1 years old). The sample included 4 men (26.7%) and 11 women (73.3%), as well as 8 right (53.3%) and 7 left eyes (46.7%), randomly selected. The different parameters and anatomical measurements of the different tests performed before contact lens fitting trials are summarized in Table 1.

**Figure 2:**
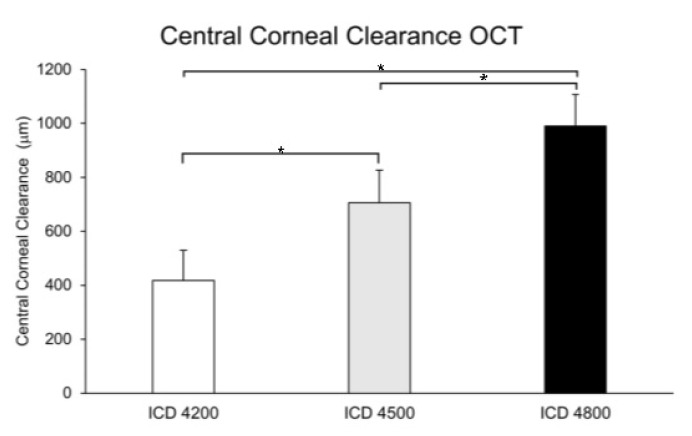
Central corneal clearance with the different scleral contact lenses (ScCL) fitted. Measurement of the corneal clearance with the ScCLs of sagittal heights 4200 µm (white), 4500 µm (grey) and 4800 µm (black). * *p* < 0.05; Wilcoxon test, Bonferroni test. Error bars represent SD.

The analysis of central corneal clearance (Figure 2) showed that the ICD lens with 4200 µm sagittal height (ICD 4200) had a significantly lower corneal clearance compared to the values of the ICD lens with 4500 µm of sagittal height (ICD 4500) and with the ICD lens with 4800 µm of sagittal height (ICD 4800) (*p* < 0.001). Mean values of corneal clearance were 418.1 ± 112.1 µm, 706.5 ± 120.3 µm and 989.9 ± 117.0 µm, respectively. In addition, there were significant differences when comparing the central corneal clearance with the ICD 4500 lens and with the ICD 4800 lens (*p* < 0.001) (Figure 2). These changes in central corneal clearance did not have a significant effect on central corneal thickness (*p* ≥ 0.569), but it should be considered that the wearing period time of each ScCL was short.

**Figure 3:**
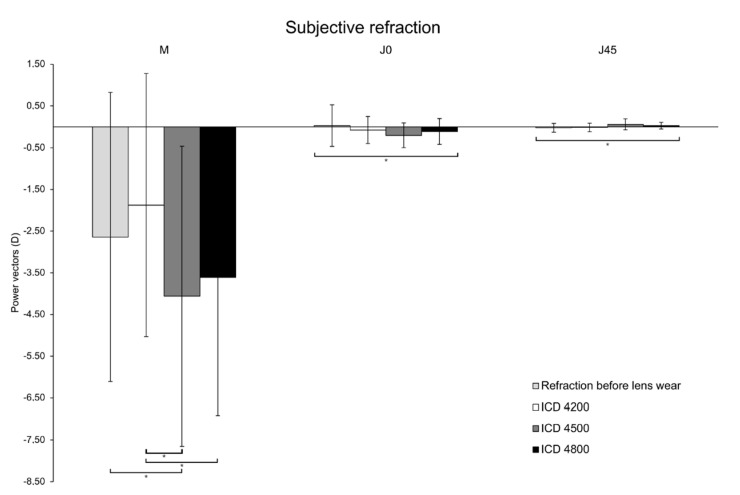
Subjective refraction obtained in before lens wear (light grey) and with the different scleral lenses (ScCL) of sagittal heights of 4200 µm (white), 4500 µm (grey) and 4800 µm (black). * *p* < 0.05; Friedman test. Error bars represent SD.

Regarding subjective refraction (Figure 3), the value of M (corresponding to the spherical equivalent) with the ScCL ICD 4500 was significantly higher (−4.06 ± 3.60 D) than the values obtained before lens wear (−2.64 ± 3.47 D, *p* = 0.006) and with the ScCL ICD 4200 (−1.88 ± 3.16 D, *p* = 0.001). Furthermore, mean M value obtained with the ICD 4800 lens was significantly higher (−3.61 ± 3.31 D) compared to the mean value obtained with ScCL ICD 4200 (*p* = 0.001). Regarding the power vectors J_0_ (*p* = 0.035) and J_45_ (*p* = 0.009) (Figure 3), significant differences between lenses and baseline conditions were found.

To evaluate the possible flexure of the three selected ScCLs, a keratometric analysis over the anterior surface of the ScCLs fitted was performed. The magnitude of the astigmatism was higher with the ScCL ICD 4500 compared to the mean values obtained with ICD 4200 and with ICD 4800 (Table 2). Differences were statistically significant (*p* = 0.047), but when applying the Bonferroni correction in the comparison by pairs, any difference was found to be associated with a *p*-value < 0.05.

**Figure 4:**
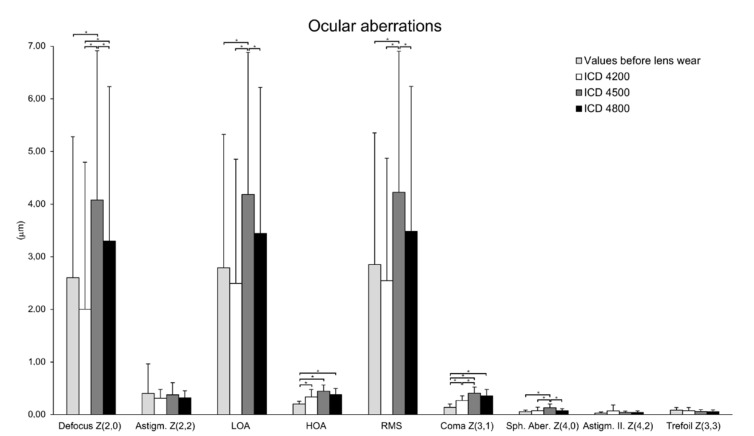
Ocular aberrations obtained before lens wear (light grey) and with the different scleral lenses (ScCL) fitted of sagittal heights 4200 µm (white), 4500 µm (grey) and 4800 µm (black). There were significant differences between the different mean values. * *p* < 0.05; Wilcoxon test, Bonferroni test. Error bars represent SD.

Some significant changes were found in ocular aberrations with the different ScCLs fitted (Figure 4). The aberrometric changes measured with the three ScCLs fitted and before fitting are shown in Table 3. Defocus, spherical aberration, low order (LOA), high order (HOA) and total root mean square (RMS) were significantly higher with ICD 4500 compared to the results obtained before lens fitting and after fitting with ICD 4200 and ICD 4800 lenses (all *p*-values < 0.007). Concerning coma, it was significantly lower before fitting (0.14 ± 0.06 μm) with respect to the values obtained with the fitting of ICD 4200 (0.27 ± 0.09 μm), ICD 4500 (0.41 ± 0.12 μm) and ICD 4800 (0.36 ± 0.12 μm) (all *p*-values = 0.001). Likewise, a significantly higher value of coma was measured with ICD 4500 compared to ICD 4200 (*p* = 0.003). These aberrometric changes had a non-significant impact on BCVA (*p* ≥ 0.384).

A significant correlation of the change in ocular coma aberration was found with the power vector J_0_ measured with the ScCL ICD 4200 (r = 0.547, *p* = 0.035). Likewise, the change in spherical aberration with the ICD 4200 lens was significantly correlated with the J_45_ power vector (r = −0.629, *p* = 0.012).

## 4. Discussion

To this date, there are some studies that have assessed the impact of ScCL vault at the level of physiology and eye health, [9,10,11,14] but never at the level of ocular optical aberrations. The closest investigation to the current study was conducted by Otchere et al. [13], who evaluated the impact of the corneal clearance on visual acuity, obtaining worse results with the ScCL fitted that generated a higher central corneal clearance (mean value of vault thickness 450 ± 170 µm). This inferior visual acuity obtained with the largest vault may be attributable to a high-order aberrometric increase, as we have observed in the current study. However, in our study, the increase in the vault that was also associated with an aberrometric increase which did not have a significant impact on visual acuity. It should be noted that the visual acuity is a parameter less sensitive to aberrometric changes than others such as contrast sensitivity or the subjective evaluation of the visual quality perceived by means of validated questionnaires. Future studies on visual quality and ScCL vault should be conducted considering these variables that truly represent what the patient notices.

ScCLs are characterized by their full bearing on the sclera, generating a corneal clearance between the lens and the corneal surface, which prevents the contact between the lens and the cornea [1,2]. Several studies have evaluated the asymmetry of the corneo-scleral profile in order to achieve an optimization of the fitting of the ScCLs, allowing the creation of a corneal clearance as symmetrical and predictable as possible. In healthy subjects, it has been shown that the sclero-conjunctival asymmetry is higher, with an increasing diameter of the area analyzed [16]. In addition, the nasal corneo-scleral angle has a more concave profile with respect to the temporal corneo-scleral angle [17]. For this reason, in this study, considering that the total diameter of the ScCL fitted was 16.5 mm, the design with toric peripheral geometry was selected in order to optimize the fitting, to reduce the decentration of the fitted lenses and to generate a more homogeneous central corneal clearance compared to a spherical geometry. The final objective was to avoid the presence of completely asymmetric post-lens meniscus that could have interfered with the results obtained. Indeed, no cases of significantly asymmetric post-lens meniscus were found in the sample evaluated.

Despite the fact that the number of ScCLs fitted has grown in recent years [18], there is no full agreement in the optimal central corneal clearance value that must be used for fitting these lenses. Indeed, central corneal clearance thickness values change from one study to another [12,19], depending on the type of ScCL fitted and the contact specialist who performed the fittings [2]. In our sample, a significant difference in the thickness of the central corneal clearance was observed after fitting several ScCLs with the same design but different sagittal height, with an increasing thickness of the corneal clearance as the sagittal height of the fitted lens increased. In any case, it should be considered that the corneal clearance also changes during the period of ScCL wear, but this is something that was not investigated in the current study. Otchere et al. [14] demonstrated that after hours of contact lens use, there is a decrease in central corneal clearance that was not dependent on the initial thickness of the corneal clearance [14]. Different studies have shown that the thickness of the corneal clearance affects oxygen transmissibility and ocular health [9,11]. It has even been estimated that to minimize corneal hypoxia, the thickness of the corneal clearance should not be greater than 200 μm [10]. However, in this study, it is the first time that the effect at the level of ocular optical quality of different thicknesses of central corneal clearance was evaluated in healthy subjects. Specifically, it has been observed that the increase in the thickness of the central corneal clearance negatively affects the subjective refraction, with an increase in the thickness of the corneal clearance generating a more myopic spherical equivalent value. This can be explained considering that an increase in the thickness of the central corneal clearance produces a more positive tear meniscus leading to a myopization of the refraction. Theoretically, it has been shown that an increase of 100 μm in the thickness of the corneal clearance produces an increase between 0.12 D and 0.5 D in the power required in a ScCL [20]. However, in our sample, there was a myopization of the subjects when increasing the sagittal height of the ScCL from 4200 to 4500 µm, but not when increasing it from 4500 to 4800 µm. This apparently contradictory outcome may be attributed to different factors, such as a potentially large level of lens flexure with this larger meniscus or even a potentially more level of central corneal flattening associated with this large quantity of tear between cornea and contact lens. It should be also noted that the lens with larger sagittal height was fitted the last and possibly there was also some previous accumulated effect of corneal molding with the previous two lenses. Although the wearing period of the three lenses fitted was short, several authors have previously reported topographic changes associated with the initial period of ScCL wear [21,22,23]. It has been previously shown that ScCL wear induces corneal changes, including a flattening of the anterior corneal surface [12,22] and a decrease in corneal thickness [23]. Likewise, in patients with keratoconus, ScCLs have shown to induce changes in different regions of the posterior corneal curvature [23].

Besides this, the keratometric analysis over the anterior surface of the ScCL also revealed some curvature changes in the contact lens that could have also contributed to modifications in the optical power of the contact lens and consequently to over-refraction. Specifically, the analysis of the astigmatism of the anterior contact lens surface in our sample suggested that an increase in the corneal clearance could be related to a greater flexure of the ScCL, since the induced astigmatism increased, but this should be confirmed in future studies analyzing more parameters. A previous study determined that the ScCL thickness influences the level of flexure experienced by the lens [24]. In our case, all the lenses used had the same central thickness of 300 μm, as indicated by the manufacturer and confirmed in this study by OCT measurements. It can be hypothesized that the scleral lens flexure could be higher, with increasing clearances, due to the fact that the high volume of the tear in the meniscus may facilitate some level of deformation of the lens with blinking, but this should be confirmed in future studies. This potential higher flexure of the lens with increasing vaults according to the astigmatic changes detected on the anterior surface of the ScCL may also be related to the significant differences found in the value of the power vectors J_0_ and J_45_ of subjective refraction measured with ICD 4500 with respect to the baseline conditions and measurements obtained with ICD 4200 and ICD 4800. Likewise, refractive astigmatic changes could be also associated with the presence of a certain level of asymmetry in the vault.

Furthermore, in the current study, the increase in central corneal clearance was associated with significant changes in HOA RMS, coma and spherical aberration. The changes shown in ocular HOAs do not seem to be related to potential changes in corneal HOAs induced as a consequence of the contact lens wear, as has been shown in previous studies. [25]. It should be considered that the ScCLs fitted in the current study were only worn during the period needed to take the measurements performed. The increase in HOAs with the increase in the central corneal clearance may potentially be related to the more relevant optical contribution of the tear film meniscus due to the increase in its thickness. In the specific case of coma, its increase with the ScCLs with higher sagittal height might be related to some level of decentration or asymmetry of the meniscus. Specifically, the largest increase for most of the HOAs evaluated was observed for the ScCL with the medium sagittal height, 4500 µm, and not for the ScCL with the largest sagittal height. This may be attributable, as was the case with changes in spherocylindrical refraction, to a potentially more marked level of lens flexure with the use of the highest sagittal height that can contribute to the compensation of some aberrations. Likewise, a potentially higher level of corneal molding associated with the thickest meniscus could have also contributed to this finding. All of these aberrometric changes associated with ScCL vault changes must be evaluated in detail in future larger samples and especially when fitting multifocal ScCLs for which the induction of spherical aberration must be controlled to enlarge effectively the depth of focus. Finally, for the SCCL of lower sagittal height, there was a correlation of spherical aberration and coma with the power vector components, suggesting a certain level of relationship between low and high order aberrometric changes.

This study has been conducted in healthy individuals with regular corneas but, as previously mentioned, one of the main indications of ScCL fitting is irregular corneas, such as keratoconus. However, the trends observed here in healthy corneas should also be similar in irregular corneas, which would be consistent with the results of a previous research demonstrating that an increased vault thickness was associated with a decrease in visual acuity measured with high and low contrast [15]. Possibly, to achieve a more optimal compensation of HOAs in these complex corneas, the use of lower vaults would be recommendable, especially when large amounts of HOAs are present. This should be investigated further in future studies including a sample of irregular corneas fitted with different sagittal heights.

Regarding the limitations of the study, the size of the sample analyzed in this study was limited, but it could be considered as a pilot study showing the trends that should be confirmed in large scale studies. Another limitation of the study is the use of the ScCLs obtained from the trial box (ICD, Family of lens designs), facilitating the study and allowing one to perform the measurements in a same session. However, these ScCLs could have been customized in each case considering the specific level of toricity of the sclera o adjusting the limbal clearance zone to achieve a best fitting to the corneo-scleral transition. Likewise, the central corneal clearance thickness obtained with the different ScCL fitted had a mean value higher than that considered as adequate (200–300 μm) [2], since these lenses are intended for fitting in cases of keratoconus that have a greater sagittal height and greater asymmetry in the corneoscleral profile compared to healthy subjects [26]. Finally, it must be noted that visual acuity and aberrations was assessed 10 min after fitting and longer time would have led to a change in corneal clearance, as demonstrated in previous studies [14]. However, the aim of the current pilot study was to demonstrate that changes in central corneal clearance of ScCLs can have a relevant optical impact. Once this has been demonstrated, future prospective trials should be conducted to investigate the real effect of changes in ScCL vault during the wearing time on ocular optical quality and how this is perceived by the patient. Therefore, this study is the initial point of a wider research area. Furthermore, it should be considered that ScCLs are mainly used in abnormal corneas, such as keratoconus or corneas after keratoplasty, with other factors affecting vision and aberrations that must be also investigated.

## 5. Conclusions

In conclusion, the increase in central corneal clearance when fitting ScCLs affects refraction, leading to a more myopic refractive error, and inducing an increase in the different ocular HOAs, especially coma and spherical aberration. This issue should be investigated further, especially the effect when fitting multifocal ScCLs, considering that these lenses induce controlled levels of spherical aberration to enlarge the depth of focus that cannot be altered by any additional element inducing aberrations.

## Figures and Tables

**Table 1:** Summary of the anatomical, refractive and ocular aberrometric measurements before lens wear in the sample evaluated.

Anatomical Measurements	Mean ± SD
Asphericity of the cornea (Q)	−0.30 ± 0.06
White-to-white distance (WTW) (mm)	12.59 ± 0.45
Central corneal thickness (CCT) (μm)	554.87± 27.77
Anterior chamber depth (ACD) (mm)	3.26 ± 0.19
Sagittal height (over a 10 mm chord) (μm)	2341.33 ± 77.54
Keratometry (K) (D)	
Flat K	42.37 ± 1.53
Curve K	43.07 ± 1.30
M (D)	−2.64 ± 3.47
J_0_ (D)	0.03 ± 0.50
J_45_ (D)	−0.02 ± 0.10
Defocus Z (2, 0) (µm)	2.60 ± 2.68
Astigmatism Z (2, ±2) (µm)	0.40 ± 0.56
LOA RMS (µm)	2.79 ± 2.54
HOA RMS (µm)	0.20 ± 0.05
Total RMS (µm)	2.86 ± 2.50
Coma Z (3, ±1) (µm)	0.14 ± 0.06
Spherical aberration Z (4, 0) (µm)	0.06 ± 0.03
Astigmatism II Z (4, ±2)	0.03 ± 0.02
Trefoil Z (3, ±3)	0.09 ± 0.05

**Table 2:** Keratometry over the anterior surface of the different ScCLs fitted.

Keratometry (K)			
Mean ± SD (D)	ICD 4200	ICD 4500	ICD 4800
Flat K	41.22 ± 0.69	43.58 ± 0.84	43.55 ± 0.40
Curved K	41.38 ± 0.71	43.87 ± 0.80	43.75 ± 0.33
Astigmatism	−0.17 ± 0.36	−0.28 ± 0.21	−0.20 ± 0.25

**Table 3:** Differences in the ocular aberrations measured with the three ScCLs fitted with respect to before fitting. * *p* < 0.05; Wilcoxon test, Bonferroni test.

Mean ± SD (µm)	ICD 4200	ICD 4500	ICD 4800
Defocus Z (2, 0)	−0.60 ± 1.30	1.48 ± 1.31 *	0.70 ± 1.29
Astigmatism Z (2, ±2)	−0.09 ± 0.51	−0.02 ± 0.45	−0.09 ± 0.54
LOA RMS	−0.30 ± 1.32	1.40 ± 1.06 *	0.66 ± 1.09
HOA RMS	0.14 ± 0.16 *	0.24 ± 0.10 *	0.18 ± 0.10 *
Total RMS	−0.31 ± 1.32	1.37 ± 1.07 *	0.63 ± 1.10
Coma Z (3, ±1)	0.13 ± 0.08 *	0.27 ± 0.11 *	0.22 ± 0.10 *
Spherical aberration Z (4, 0)	0.02 ± 0.08	0.07 ± 0.07 *	0.02 ± 0.06
Astigmatism II Z (4, ±2)	0.04 ± 0.12	0.01 ± 0.03	0.01 ± 0.04
Trefoil Z (3, ±3)	−0.01 ± 0.09	−0.03 ± 0.06	−0.03 ± 0.06

## Data Availability

Not applicable.
